# Functional Conservation of *Cis*-Regulatory Elements of Heat-Shock Genes over Long Evolutionary Distances

**DOI:** 10.1371/journal.pone.0022677

**Published:** 2011-07-25

**Authors:** Zhengying He, Kelsie Eichel, Ilya Ruvinsky

**Affiliations:** Department of Ecology and Evolution, Institute for Genomics and Systems Biology, The University of Chicago, Chicago, Illinois, United States of America; Centre for Genomic Regulation (CRG), Universitat Pompeu Fabra, Spain

## Abstract

Transcriptional control of gene regulation is an intricate process that requires precise orchestration of a number of molecular components. Studying its evolution can serve as a useful model for understanding how complex molecular machines evolve. One way to investigate evolution of transcriptional regulation is to test the functions of *cis*-elements from one species in a distant relative. Previous results suggested that few, if any, tissue-specific promoters from Drosophila are faithfully expressed in *C. elegans*. Here we show that, in contrast, promoters of fly and human heat-shock genes are upregulated in *C. elegans* upon exposure to heat. Inducibility under conditions of heat shock may represent a relatively simple “on-off” response, whereas complex expression patterns require integration of multiple signals. Our results suggest that simpler aspects of regulatory logic may be retained over longer periods of evolutionary time, while more complex ones may be diverging more rapidly.

## Introduction

Expression patterns of some genes appear to be highly conserved even between distantly related species [1]–[3]. One possible explanation for this observation is that *cis*-regulatory sequences retain their functions over long periods of evolutionary time. Sequence comparisons alone are unable to reveal whether orthologous *cis*-regulatory elements are functionally conserved. This is due to the fact that we are unable to deduce the spatio-temporal expression patterns from the primary sequence of putative promoters and enhancers [4]. In at least some instances, regulatory elements that retain little recognizable sequence conservation can direct similar expression patterns [5]. Thus, presently experimentation is the only way to establish whether *cis*-regulatory elements are functionally conserved between species, that is, whether a promoter can drive an expression pattern similar to its endogenous pattern, when placed in a different species.

A systematic survey of *cis*-regulatory elements from Drosophila suggested that few, if any, of them functioned properly when placed in *C. elegans* [6]. Some directed little or no expression, while others were expressed in inappropriate patterns, e.g. neuronal enhancers driving gene expression in muscles. These results may indicate that the phylogenetic distance between flies and worms is too large for functional conservation of any promoters. Alternatively, distinct types of *cis*-regulatory elements may be evolving under different regimes. The majority of the *cis*-elements tested in swaps among distant species were from genes expressed in relatively narrow tissue-specific patterns. Therefore, the results to date may reflect the peculiar nature of these genes and may not be generalizable to all genes.

One type of genes that was not represented in the systematic functional survey of Drosophila *cis*-elements in *C. elegans* were stress-induced genes such as those encoding heat-shock proteins. To test whether *cis*-regulatory elements of these genes retained functional conservation for longer periods of time than promoters of tissue-specific genes, we examined expression patterns directed by Drosphila and human promoters of several stress-induced genes in *C. elegans*.

## Results and Discussion

### Promoters of Drosophila and human heat-shock genes are activated in *C. elegans*

When placed in adverse environments, organisms activate an elaborate defense mechanism known as the heat-shock response [7], that is characterized by increased transcription of heat-shock proteins [8]. We tested whether promoters of Drosophila and human heat-shock protein genes can drive increased expression when placed in *C. elegans*. To characterize temporal and spatial patterns of transcription in response to heat shock we used constructs fusing promoters to reporter genes (see Materials and Methods and Supporting Information for details).

**Figure 1:**
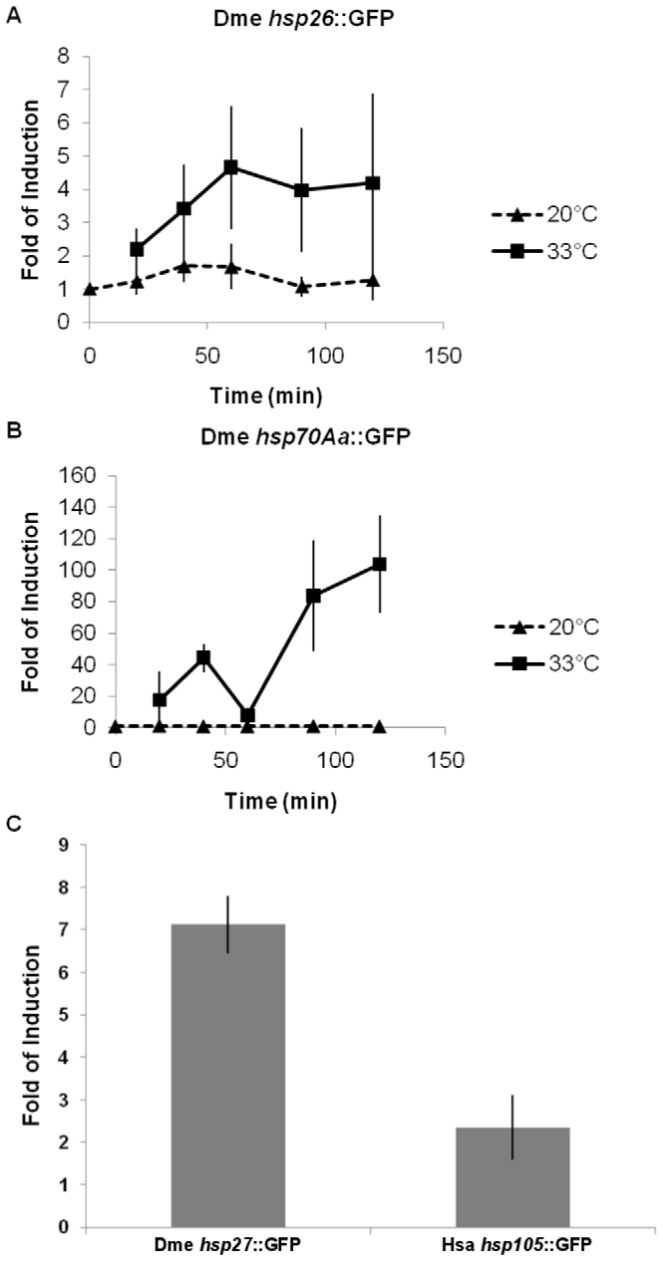
Induction of promoters of Drosophila and human heat-shock genes in *C. elegans*. A) Worms carrying reporter transgenes were heat shocked at 33°C for the indicated periods of time. The mRNA level was measured by quantitative RT-PCR. Each data point represents 3 independent biological replicates (with standard error). B) Same as in A), except each data point represents 2 independent biological replicates. C) Worms carrying reporter transgenes were heat shocked at 35°C, and 30–40 minutes later the mRNA level were measured by quantitative RT-PCR in 2 independent biological replicates.

We selected three Drosophila genes: *hsp26* [9]–[11], *hsp70Aa* [10], [12], [13], *hsp27* [14], [15], and a human gene *hsp105* [16], [17]. Reporter constructs fusing promoters (these were defined in previously published experimental studies) of the first two genes to GFP showed induction profiles characteristic of endogenous heat-shock gene activation (Figure 1A, B), although the highest fold of induction in both cases was lower than that seen in the endogenous *trans*-regulatory environment (compare Figure 1 and Table 1). The remaining two promoters were induced by heat shock (Figure 1C), although exposure to a higher temperature (35°C, not 33°C) was required to obtain consistent results. We also tested whether promoter of a *S. cerevisiae* heat-shock gene *ssa3* [18] was inducible in *C. elegans* but failed to detect any evidence of heat-induced activation (data not shown).

**Table 1:** Heat-shock inducible genes examined in this study.

Species	Gene	Promoter length	Endogenous induction	Endogenous expression
*D. melanogaster*	hsp26	696 bp	90 fold	Spermatocytes, nurse cells, epithelium, imaginal discs, proventriculus and neurocytes
*D. melanogaster*	hsp70Aa	783 bp	200 fold	Third instar lavae. No expression without heat shock. Rapid induction in brain, salivary glands, imaginal disks and hindgut
*D. melanogaster*	hsp27	605 bp	14 fold	Early larval brain and gonads, imaginal discs of early pupae, adult central nervous system and germline
*S. cerevisiae*	ssa3	1117 bp	20 fold	N/A
*H. sapiens*	hsp105	1398 bp	28 fold	N/A

**Figure 2:**
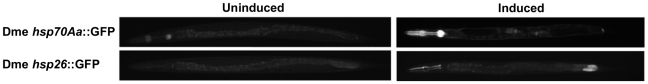
Expression patterns in *C. elegans* of Drosophila promoters of heat-shock genes fused to GFP. Worms were heat shocked at 33°C for 1 hour and allowed to recover at 20°C for 6–7 hours. Images are composites adjusted for exposure and taken in different planes.

We next examined whether, when placed in *C. elegans*, promoters of Drosophila heat-shock genes are activated in spatial patterns comparable to the sites of their endogenous expression. The *cis*-elements that regulate inducibility may be separate from those conferring tissue-specific expression [11], [19], [20]. We examined the two constructs that showed the strongest and most consistent induction – Drosophila *hsp70Aa* and *hsp26* (Figure 2). Upon heat shock, both were strongly upregulated in the pharynx and, to some extent, the intestine. Although the latter may represent an overlap with the sites of endogenous expression, expression in other tissues, most notably the nervous system, was conspicuously missing (compare with Table 1). We interpret these differences as an indication that, in *C. elegans*, some components of the *trans*-regulatory environment are either missing from certain cell types (in this case neurons) or have functionally diverged to an extent that they are no longer able to activate expression from Drosophila heat-shock promoters.

### Implications for understanding the evolution of transcriptional regulation

Our data suggest that promoters of all four tested Drosophila and human heat-shock genes retain the ability to be induced even in the context of a highly divergent *trans*-regulatory environment of a *C. elegans* as a host organism. Previously, it has been shown that a promoter of the Drosophila *hsp70* retains inducibility when placed into other divergent species [21]–[23]. These results stand in stark contrast to the tests of Drosophila promoters of tissue-specific [6] and possibly housekeeping (Figure S1) genes. In those cases little or no expression was seen for the majority of promoters and none that can be reasonably interpreted as conserved patterns.

Three explanations, which are not mutually exclusive, can account for our findings. First, different criteria are used to define “conservation” for promoters of tissue-specific and heat-shock genes. The former would be required to be expressed in similar spatial pattern (or at least in homologous cell types) in both compared species. In contrast, the latter would “only” need to be induced by stress, without conservation of pattern. When Drosophila heat-shock promoters are considered with regard to the spatial patterns of expression in *C. elegans*, they failed to recapitulate the correct pattern, a result that is no different from tissue-specific promoters [6].

Second, stress-response networks appear to be highly conserved [24]. This certainly applies to HSF, the heat-shock transcription factor responsible for induction of heat-shock gene expression [25], [26]. A human ortholog is able to rescue a *S. cerevisiae* HSF mutant [27] and a Drosophila ortholog can rescue a *S. pombe* mutant [28], despite some functional divergence [29]. It is conceivable that the highly conserved nature of the HSF protein has contributed to the extraordinary level of functional conservation of heat-shock gene promoters. This explanation alone, however, does not appear to be sufficient as functions of other transcription factors are highly conserved between distantly related species [30], [31].

Finally, appropriate tissue-specific patterns of gene expression are achieved by coordinated action of multiple independent transcription factors binding to *cis*-elements [4], [32]. In many instances, this requires a particular *cis*-regulatory architecture, that is, the relative number, location and spacing of transcription factor binding sites [33]. Functional integrity of diverging orthologous *cis*-sequences is assured by the coevolution within *cis*-elements [34]–[36], between transcription factors [37], [38], and between transcription factors and their binding sites [39]. Over extended periods of time, such as that separating the nematode and arthropod lineages, enough changes must accumulate to render most Drosophila enhancers “unintelligible” to *C. elegans* transcriptional machinery.

In contrast, transcriptional activation of heat-shock genes is mediated by the binding of the heat-shock transcription factor (HSF) to HSF binding elements (HSEs) within promoters [40]. The presence of HSE sites in heat-shock promoters is a major determinant of inducibility, although other factors also influence levels of induction [10], [41] and even whether a particular promoter is a target of HSF [42]. We searched for occurrences of a motif previously defined as a binding site of *C. elegans* HSF [43] in promoter sequences of Drosophila and human promoters tested here (Figure S2). We found strong matches to consensus motifs in all promoters except for *hsp27*. In Drosophila genes many motifs overlapped with previously annotated HSEs [44]. If the presence of HSEs in promoters is highly constrained during evolution, and if their presence is sufficient for inducibility [41], the *cis*-regulatory elements of heat-shock genes may retain functional conservation for long periods of time. We propose that the elements of transcriptional gene regulation, such as inducibility, that are controlled by “simpler” regulatory logic may retain functions over longer periods of time. In contrast, promoters that integrate multiple signals undergo relatively rapid turnover compensated by coevolving transcription factors and *cis*-regulatory sequences.

## Materials and Methods

### Plasmids and worm strains

Putative *cis*-regulatory regions were PCR amplified from genomic DNA of relevant species. Worm genomic DNA was extracted from *C. elegans* N2 strain or *C. briggsae* AF16 strain. Drosophila DNA was a gift of Cecilia Miles (The University of Chicago). Human genomic DNA was obtained from Clontech (catalog # 636401). The PCR products were cloned upstream of GFP (vector pPD95.75) or mCherry (vector pPD95.79) reporter genes. Constructs were sequenced before injections (complete sequences are shown in Figure S2). The Hsa hsp105::GFP and Dme hsp27::GFP constructs were injected at 0.5 ng/µl because injections at higher concentrations appeared to cause lethality. All other constructs were injected at 5 ng/µl. Constructs were co-injected with a *pha-1* rescuing construct (at 10 ng/µl) into *C. elegans pha-1* (*e2123*) strain [45]. Because this *pha-1* mutation is a conditional lethal, all surviving progeny can be presumed to be transgenic. In cases when reporter gene expression was not detected, we further verified by PCR that worms did indeed carry appropriate transgenic constructs. Dozens of individuals from multiple independent strains were examined to ensure consistency. Photographs were taken on a Leica DM5000B compound microscope.

### Heat shock

Gravid worms were bleached. The newly hatched L1 larvae were placed on NGM plates seeded with OP50 bacteria and allowed to grow at 20°C for 46 to 50 hours. 30 to 100 worms were then transferred to OP50-seeded NGM plates that were then placed at the heat-shock temperature or 20°C (controls) for the indicated time periods. This was followed by a 20-minute recovery at 20°C. Next, the worms were washed off the plates with M9 solution and pelleted by centrifugation. The worms were then washed twice with M9 and snap-frozen.

### RNA and DNA extraction, and quantitative RT-PCR

Total RNA was extracted using PrepEase® RNA Spin Kit (USB, catalog #78767). The manufacturer's protocol was slightly modified: 350 µl buffer RA1 and 4 µl β-ME were added to each sample containing 30–200 worms, vortexed for 1 minute, and subjected to 4 cycles of snap-freezing and thawing. Samples were vortexed for 30 to 60 minutes and purified as described in the manufacturer's protocol. mRNA was reverse transcribed with the iScript™ cDNA Synthesis Kit (Bio-Rad catalog #170-8891). Worm DNA was extracted with the DNeasy Blood & Tissue Kit (Qiagen, catalog #59504). qPCR was done using either the SYBR® Advantage® qPCR Premix (Clontech, catalog #639676) or the HotStart-IT® SYBR® Green qPCR Master Mix (USB, catalog #75762) using ABI 7900HT Fast Real-Time PCR System. Expression levels of reporter constructs were normalized to endogenous non-inducible (Figure S3) genes *act-2* (actin, WBGene00000064) and *gpd-2* (glyceraldehyde-3-phosphate dehydrogenase, WBGene00001684). Relative levels of induction were calculated based on the amount of expression just prior to the start of heat-shock treatment.

### Experimental controls

As a positive control, we verified that promoters of *C. elegans* and *C. briggsae* heat-shock genes can drive increased expression upon heat shock when fused to reporter genes. We tested promoters of *C. elegans hsp-70* (WBGene00002026) and its *C. briggsae* counterpart (WBGene00040668). As shown by quantitative reverse transcription followed by PCR (qRT-PCR), expression from the endogenous loci of these genes was induced (∼70 to ∼300 fold) within 10 minutes from exposure to heat (30°C; Figure S4). Constructs fusing promoters of these two genes to GFP were injected in *C. elegans*. Strains carrying these constructs displayed induction of expression upon heat shock (Figure S4), which was qualitatively consistent with induction profiles of endogenous genes. As a negative control, we showed that expression of transgenes fusing mCherry or GFP to promoters of genes not known to be heat-induced (*myo-2* and *unc-47*) remained unchanged after a heat shock (Figure S3). Taken together, these experiments demonstrate that transgenic nematodes carrying promoter fusions to reporter genes could capture, at least qualitatively, the ability of a promoter to be induced under conditions of heat shock.

## Supporting Information

Figure S1 **Expression pattern in** ***C. elegans*** **of the Drosophila promoter of housekeeping gene** ***Gapdh2*** **fused to GFP.**
(PDF)

Figure S2 **Sequences of** ***cis*** **-regulatory elements tested in this study.** Species, names of the genes and the length of inserts are indicated as well as whether these were fused to GFP or mCherry.
(DOC)

Figure S3 **Controls.** A) Endogenous expression of *C. elegans* genes *act-2* and *gpd-2* is not induced after heat shock. Expression of promoter-reporter gene constructs B) *myo-2*::mCherry and C) *unc-47*::GFP is not induced after heat shock.
(PDF)

Figure S4 **Induction by heat shock of endogenous** ***C. elegans*** **and** ***C. briggsae hsp-70*** **genes and of transgenic constructs fusing their promoters to GFP.** Relative levels of induction were calculated based on the amount of expression just prior to the start of heat-shock treatment.
(PDF)

## References

[1] Shubin N, Tabin C, Carroll S (2009). Deep homology and the origins of evolutionary novelty.. Nature.

[2] Fukushige T, Brodigan TM, Schriefer LA, Waterston RH, Krause M (2006). Defining the transcriptional redundancy of early bodywall muscle development in C-elegans: Evidence for a unified theory of animal muscle development.. Genes Dev.

[3] Avidor-Reiss T, Maer AM, Koundakjian E, Polyanovsky A, Keil T (2004). Decoding cilia function: Defining specialized genes required for compartmentalized cilia biogenesis.. Cell.

[4] Wray GA, Hahn MW, Abouheif E, Balhoff JP, Pizer M (2003). The evolution of transcriptional regulation in eukaryotes.. Mol Biol Evol.

[5] Hare EE, Peterson BK, Iyer VN, Meier R, Eisen MB (2008). Sepsid even-skipped enhancers are functionally conserved in drosophila despite lack of sequence conservation.. Plos Genetics.

[6] Ruvinsky I, Ruvkun G (2003). Functional tests of enhancer conservation between distantly related species.. Development.

[7] Lindquist S (1986). The heat-shock response.. Annu Rev Biochem.

[8] Morimoto RI (1993). Cells in stress - transcriptional activation of heat-shock genes.. Science.

[9] Sandaltzopoulos R, Mitchelmore C, Bonte E, Wall G, Becker PB (1995). Dual regulation of the drosophila Hsp26 promoter in-vitro.. Nucleic Acids Res.

[10] Simon JA, Lis JT (1987). A germline transformation analysis reveals flexibility in the organization of heat-shock consensus elements.. Nucleic Acids Res.

[11] Glaser RL, Wolfner MF, Lis JT (1986). Spatial and temporal pattern of Hsp26 expression during normal development.. EMBO J.

[12] Shilova VY, Garbuz DG, Myasyankina EN, Chen B, Evgen'ev MB (2006). Remarkable site specificity of local transposition into the HsP70 promoter of drosophila melanogaster.. Genetics.

[13] Ishhorowicz D, Pinchin SM, Schedl P, Artavanistsakonas S, Mirault ME (1979). Genetic and molecular analysis of the 87a7 and 87c1 heat-inducible loci of D melanogaster.. Cell.

[14] Pauli D, Tonka CH, Tissieres A, Arrigo AP (1990). Tissue-specific expression of the heat-shock protein Hsp27 during drosophila-melanogaster development.. J Cell Biol.

[15] Riddihough G, Pelham HRB (1986). Activation of the drosophila-Hsp27 promoter by heat-shock and by ecdysone involves independent and remote regulatory sequences.. EMBO J.

[16] Trinklein ND, Chen WC, Kingston RE, Myers RM (2004). Transcriptional regulation and binding of heat shock factor 1 and heat shock factor 2 to 32 human heat shock genes during thermal stress and differentiation.. Cell Stress Chaperones.

[17] Ishihara K, Yasuda K, Hatayama T (1999). Molecular cloning, expression and localization of human 105 kDa heat shock protein, hsp105.. Biochimica Et Biophysica Acta-Gene Structure and Expression.

[18] Boorstein WR, Craig EA (1990). Transcriptional regulation of Ssa3, an Hsp70 gene from saccharomyces-cerevisiae.. Mol Cell Biol.

[19] Lakhotia SC, Prasanth KV (2002). Tissue- and development-specific induction and turnover of hsp70 transcripts from loci 87A and 87C after heat shock and during recovery in drosophila melanogaster.. J Exp Biol.

[20] Cohen RS, Meselson M (1985). Separate regulatory elements for the heat-inducible and ovarian expression of the drosophila Hsp26 gene.. Cell.

[21] Mcmahon AP, Novak TJ, Britten RJ, Davidson EH (1984). Inducible expression of a cloned heat-shock fusion gene in sea-urchin embryos.. Proceedings of the National Academy of Sciences of the United States of America-Biological Sciences.

[22] Mirault ME, Southgate R, Delwart E (1982). Regulation of heat-shock genes - a dna-sequence upstream of drosophila-Hsp70 genes is essential for their induction in monkey cells.. EMBO J.

[23] Corces V, Pellicer A, Axel R, Meselson M (1981). Integration, transcription, and control of a drosophila heat-shock gene in mouse cells.. Proceedings of the National Academy of Sciences of the United States of America-Biological Sciences.

[24] Kirienko NV, Fay DS (2010). SLR-2 and JMJC-1 regulate an evolutionarily conserved stress-response network.. EMBO J.

[25] Vuister GW, Kim SJ, Orosz A, Marquardt J, Wu C (1994). Solution structure of the dna-binding domain of drosophila heat-shock transcription factor.. Nat Struct Biol.

[26] Wiederrecht G, Shuey DJ, Kibbe WA, Parker CS (1987). The saccharomyces and drosophila heat-shock transcription factors are identical in size and dna-binding properties.. Cell.

[27] Liu XD, Liu PCC, Santoro N, Thiele DJ (1997). Conservation of a stress response: Human heat shock transcription factors functionally substitute for yeast HSF.. EMBO J.

[28] Gallo GJ, Prentice H, Kingston RE (1993). Heat-shock factor is required for growth at normal temperatures in the fission yeast schizosaccharomyces-pombe.. Mol Cell Biol.

[29] Sakurai H, Takemori Y (2007). Interaction between heat shock transcription factors (HSFs) and divergent binding sequences - binding specificities of yeast HSFS and human HSF1.. J Biol Chem.

[30] Westmoreland JJ, McEwen J, Moore BA, Jin YS, Condie BG (2001). Conserved function of caenorhabditis elegans UNC-30 and mouse Pitx2 in controlling GABAergic neuron differentiation.. Journal of Neuroscience.

[31] Grens A, Mason E, Marsh JL, Bode HR (1995). Evolutionary conservation of a cell fate specification gene: The hydra achaete-scute homolog has proneural activity in drosophila.. Development.

[32] Halfon MS, Carmena A, Gisselbrecht S, Sackerson CM, Jimenez F (2000). Ras pathway specificity is determined by the integration of multiple signal-activated and tissue-restricted transcription factors.. Cell.

[33] Crocker J, Tamori Y, Erives A (2008). Evolution acts on enhancer organization to fine-tune gradient threshold readouts.. Plos Biology.

[34] Shaw PJ, Wratten NS, McGregor AP, Dover GA (2002). Coevolution in bicoid-dependent promoters and the inception of regulatory incompatibilities among species of higher diptera.. Evol Dev.

[35] McGregor AP, Shaw PJ, Hancock JM, Bopp D, Hediger M (2001). Rapid restructuring of bicoid-dependent hunchback promoters within and between dipteran species: Implications for molecular coevolution.. Evol Dev.

[36] Ludwig MZ, Bergman C, Patel NH, Kreitman M (2000). Evidence for stabilizing selection in a eukaryotic enhancer element.. Nature.

[37] Li VC, Davis JC, Lenkov K, Bolival B, Fuller MT (2009). Molecular evolution of the testis TAFs of drosophila.. Mol Biol Evol.

[38] Liu Q, Nakashima-Kamimura N, Ikeo K, Hirose S, Gojobori T (2007). Compensatory change of interacting amino acids in the coevolution of transcriptional coactivator MBF1 and TATA-box-binding protein.. Mol Biol Evol.

[39] Athanikar JN, Osborne TF (1998). Specificity in cholesterol regulation of gene expression by coevolution of sterol regulatory DNA element and its binding protein.. Proc Natl Acad Sci U S A.

[40] Akerfelt M, Morimoto RI, Sistonen L (2010). Heat shock factors: Integrators of cell stress, development and lifespan.. Nature Reviews Molecular Cell Biology.

[41] Fernandes M, Xiao H, Lis JT (1995). Binding of heat shock factor to and transcriptional activation of heat shock genes in drosophila.. Nucleic Acids Res.

[42] Guertin MJ, Lis JT (2010). Chromatin landscape dictates HSF binding to target DNA elements.. Plos Genetics.

[43] GuhaThakurta D, Palomar L, Stormo GD, Tedesco P, Johnson TE (2002). Identification of a novel cis-regulatory element involved in the heat shock response in caenorhabditis elegans using microarray gene expression and computational methods.. Genome Res.

[44] Tian S, Haney RA, Feder ME (2010). Phylogeny disambiguates the evolution of heat-shock cis-regulatory elements in drosophila.. Plos One.

[45] Granato M, Schnabel H, Schnabel R (1994). Pha-1, a selectable marker for gene-transfer in C-elegans.. Nucleic Acids Res.

