# Supplementary material for: Functional Conservation of Cis-Regulatory Elements of Heat-Shock Genes over Long Evolutionary Distances
Source: PLoS One. 2011 Jul 25;6(7):e22677. doi: 10.1371/journal.pone.0022677 (PMC3143172; doi:10.1371/journal.pone.0022677)
Supplement: Figure S1 — Expression pattern in C. elegans of the Drosophila promoter of housekeeping gene Gapdh2 fused to GFP. (PDF) [file pone.0022677.s001.pdf]

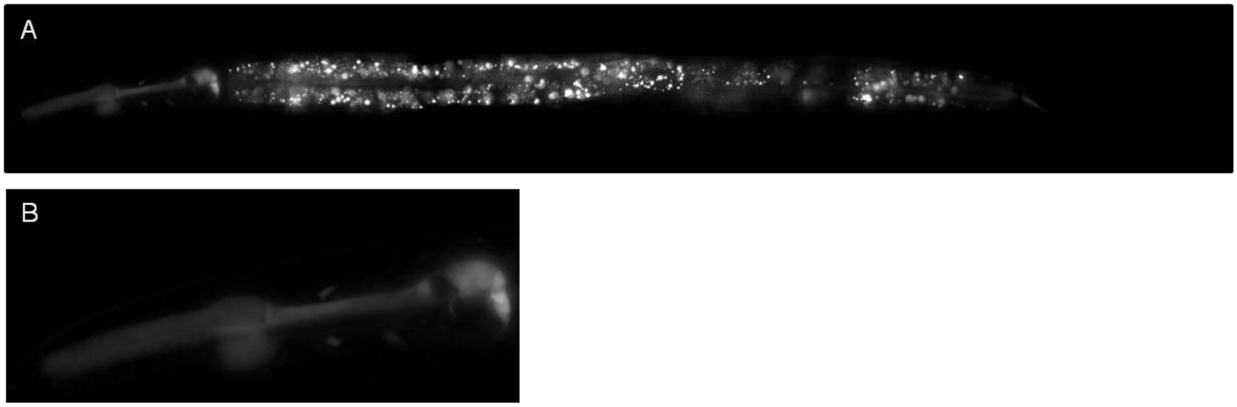

Figure S1

Figure S1. Expression pattern in *C. elegans* of the Drosophila promoter of a housekeeping gene *Gapdh2* fused to GFP.

We generated a GFP fusion to a promoter of Drosophila *Gapdh2* (Glyceraldehyde 3-phosphate dehydrogenase, CG8893) gene. This promoter has been previously tested in Drosophila (Sun XH, Lis JT, Wu R. (1988) The positive and negative transcriptional regulation of the drosophila gapdh-2 gene. Genes Dev 2(6): 743-753). *Gapdh2* is a key enzyme in glycolysis and is broadly expressed in Drosophila. Therefore, if its promoter drove a broad expression across different tissues in *C. elegans*, we would conclude that this *cis*-element was functionally conserved between worms and flies. Instead, multiple strains carrying this construct showed consistent expression in the pharynx and a few head neurons (90/100 examined animals) and to a lesser extent (20/100) in several tail neurons. In all cases, expression was relatively weak. Long exposure resulted in the considerable intestinal autofluorescence caused by the presence of pigment lipofuscin (seen as granular staining in the intestine). This pattern is not consistent with promoter function being conserved. We saw no specific expression with any other Drosophila promoters (data not shown). Panel (B) shows the same animal as in (A) but at a higher magnification. Images are composites adjusted for exposure and taken in different planes.
