# Supplementary material for: Functional Conservation of Cis-Regulatory Elements of Heat-Shock Genes over Long Evolutionary Distances
Source: PLoS One. 2011 Jul 25;6(7):e22677. doi: 10.1371/journal.pone.0022677 (PMC3143172; doi:10.1371/journal.pone.0022677)
Supplement: Figure S2 — Sequences of cis -regulatory elements tested in this study. Species, names of the genes and the length of inserts are indicated as well as whether these were fused to GFP or mCherry. (DOC) [file pone.0022677.s002.doc]

**Figure S2.**

Sequences of *cis*-regulatory elements tested in this study. Species, names of the genes and the length of inserts are indicated as well as whether these were fused to GFP or mCherry. Lower case text represents promoter sequences and caps are coding sequences. Primers are underlined, and restriction enzyme cites used for cloning are boxed.

Highlighted in yellow are motifs corresponding to *C. elegans* HSE as defined by GuhaThakurta D, Palomar L, Stormo GD, Tedesco P, Johnson TE, Walker DW, Lithgow G, Kim S, Link CD (2002) Identification of a Novel *cis*-Regulatory Element Involved in the Heat Shock Response in *Caenorhabditis elegans* Using Microarray Gene Expression and Computational Methods. Genome Res 12: 701-12.

In promoters of Drosophila heat-shock genes, letters shown in different colors correspond to HSEs as annotated by Tian S, Haney RA, Feder ME (2010) Phylogeny Disambiguates the Evolution of Heat-Shock *cis*-Regulatory Elements in *Drosophila.* PLoS ONE 5:e10669. Numbers underneath blocks of sequence represent relative position to transcription start site. The number of HSEs in each block is also given.

/*D. melanogaster*/ hsp26 (CG4183)::GFP (707 bp)

aagctttgtgacaacaactacaagtattccagtaaaacttaaagacagaaacacgaaataatgtacttaataaagaggaaaaccagaataaaaaaaactgacgttttgttttgtttgccgttagccggctgtttcttttgcgctc**tttctagaaa**attgcaacaac**tctctagaaacttcg**gctctctcactcatacaggcgcactagctctgcttttgcgcgtacgacaacaactacatttaaaatttctcgaaactcatggcatttattgggaaaggttagttagttttattttttgtttttagagcagcattcaatttagacttttataaaagaaatttctaatttgatccctcgtttatcaaacgatacaaagctatattcataattttttctctctgtgcacgttctctctcttctcttctctctctctactctttcctt**tttctgtcactttccggactcttctagaaa**agctccagcgggtataaaagcagcgtcgcttgacgaacagagcacagatcgaattcaaaaatcgagcagtgaacaactcaaagcaactttgcgcaaaagcaaaacttcaaacgagaaaaaaaaggattaaaaacctttgcttacaagtcaaacaagttcattcaacttaaccaaagaaaaaatatttcaatctcgcaaaaggaacaftaacctaaaggaaacgtaaaaATGTCGCTATCACCGGTAGAAAAAATGAGT

**-373 -364 2**

**-352 -338 3**

**-73 -44 6**

/*D. melanogaster*/ hsp70Aa (CG31366)::GFP (794 bp)

Aagcttcaacttatttcacagtgtaaacagttgacaacaacagtcttgacaacctttacgtaattttaaataaaaaacactaacaatcatctgcatgcaattgtctgtattaatctaataaataaatagcttttttaagttagtatgtaaatacatttttaagaatatcttgtcaaagttccataggcctttctggcggacaacatccgcgtaacaaacccttcgattatctctaacataattaacttaagcagccgtatttataaagaaatttccaaaataaag**cgaatattctagaat**cccaaaacaaactggttgttgcggtaggtcatttgtttggcagaa**agaaaactcgagaaatttct**ctggccgttattctctattcgttttgtgactctccctctttgtactattgctctctcactctgtcacacagtaaacggcgcact**gttctcgttgcttcgagaga**gcg**cgcctcgaatgttcgcgaaa**agagcgccggagtataaatagaggagcttcgtcgacggagagtcaattctattcaaacaagtaaagtgaacacatcgctaagcgaaagctaagcaaacaaacaagcgcagctgaacaagctaaacaatctgcaataaagtgcaagttaaagtgaatcaattaaaagtaaccaacaaccaagtaattaaactaaaaactgcaactactgaaatcaaccaagaagtaattattgaagacaagaagagaactctgaatactttcaacaagtcgttaccgaggaagaagaactcacacacaATGCCTGCTATACCGGTAGAAAAAATGAGT

**-252 -238 3**

**-192 -173 4**

**-88 -69 4**

**-65 -46 4**

/*D. melanogaster*/ hsp27 (CG4466)::GFP (619 bp)

ggatccgagcgataagagaagaaaatgctttaaataaatacatatatctgcatatatacgtacatgtacatacatatgtatgtactgcattttaactgttcgttttgctttttattcgcaaa**gagaaactccc**cagaaa**agaaatgtcaagaagtttctggttctttct**ccctctctctatgaaaagccggct**gtgctagaaagagccagaagatgcgagaga**aaactgtttgttgaattacggggcgtattcaaaggggcttttaaatgtcgcttaaattttaagtttgacaggctaataattgcttgcctatatctaaatattattatatttgcattaggggatcatagggaaaaccttctctgcaggcaaaatctaacgaagatggcaaccccccatcattttattaaagttccgtccctggttgccatgcactagtgtgtgtgagcccagcgtcagtataaaagccggcgtcaacgtcgcccgagcacagtctaaactgaaaaattgaaggcaaacgttgaagcaaacttcgctaaaaaaattcgaaaaagcaaaaaaaattcctttgtctagacagggttgtgaataaagagaaaaaaaatcaaaaATGTCAATTATACCACCGGTAGAAAAAATGAGT

**-369 -360 2**

**-353 -324 6**

**-299 -270 6**

/*D. melanogaster*/ gapdh2 (CG8893)::GFP (932 bp)

ggatcctggatgcctacttgagctgtcaggtgtttttcattttaacaatgggatgacaacagcacaattgcttaaatgaaatcatcaaaacggataatttaattacttagacaattatttcatctaatattgtagttatgcatagaagatatatacatatttacatgtatatgtatctttgtacatatgtacatgtattgtacagattaatgtcacgtaccggcgaaacttcaatttgctcagaatatgacctctaaattttaatattaatgaaattgatgaatatgtatttatgtggggatgttaggatttttccatttgcatttacttatgtacatatttatgtacaatataaccaattgatgttcatcagacgtgctcatatatgtgtatgtttcgtgtgcctatatatgcacgtatgtaattgccggcaactaagctcacaagaaagctcaacttccgtcaacaaaattgagcaggtgaaatggccataaaaatatcccaataaacatttgtccgtataaattaaaaaatccatatactttataggtcactttaactgtctaagatcagaagtttttaaagttaagcgtatcttatgtacatatctacttattatattcatataatgatgcttgcaaaaagtctattgggtagcattgagctttcacgttactgagagcaaaagctcttttttattgcggtagccgctctcgagcttgacatgcacgtccttcatctgactttgaacaatttttcgcccgagttttcgcccatagaaagcgctcaaaatttctcagccatcacagtcgattccttgcaagcaagccgataggtagtgtttccctcaattccgcggaaaactttccaagtactaactgctttacatatacatacattactttccagataaacaaaaagttaaccATGTCGAAGATACCGGTAGAAAAAATGAGT

/*S. cerevisiae*/ ssa3::GFP (1117 bp)

ggatcctggcctttacaaaatgatggaagaaagggatggcgcaaaattcgagaatattgttcacaacttcaaggaacggcagatgatggtttcttatccgaaaattgacgaagatgatacctggtacaatcttaccgagtttgtgcagatggataaaatccgaaagatagtaaggaaagatgaaaaccagttctcttacgtagattcttcgatgaccacagttcaagaaaatgagctgctaaaatccagcttgcaaaaagcaggttctaaaatggaagccaagaatgaagatgatcctgcacattctttaaactatacagtaataaacttcaaatctagagaagccataaggcctggccatgaaatggaggattttttagacaagtcttactacttgaacactgtaatgctacaaggaatttttaaaaattcaagtaattattttggggagttgcagtttgcgttcttaaatgccatgttttttggtaactacgggtcgagtttgcaatggcatgctatgatcgaactgatatgttcaagcgctacggtgcctaaacatatgctcgataaattagacgaaatcttatattatcagataaagacattgcctgaacaatactcagacatcttgttgaatgaacgagtttggaatatttgtctgtattcgtcatttcaaaaaaactccctacacaacacagaaaagataatggaaaacaaatatccagaattgcttggtaaagacaatgaagacgacgctcttatttacggtatcagtgatgaagaaagggatgacgaggatgatgagcacaaccctaccattgttggcggtctctattaccaaaggccataacgatcatcgtgcggcgctatcatcaaacgtatttgacttgatgcctatggaggttatgggtgcccttaattagggatcgctgtggaaagttatagaatattacagaagcagccacaagggtgaccagaagatggttaagggatgtatcatattgcacaattggaaacgaatggaagggtatataaagtgactgaaattggtagcataaacattcttgtatgtcaatgtttgtcactaaacggatagaataggtactaaacgctacaaagaaaaaccggtagaaaaaATGAGT

/*H. sapiens*/ hsp105::GFP (1409 bp)

ggatcctaagttatcagcaacacagttccaccttttcttacactgtctctttactgaacaaaagttaacggggacatcgcttctgagggaaagaaacaaaagctgcttcagatcaaacaaaaagcaaaacttacacacagtaccaatttatggctaggagtggcagggtagaacagcgtcggcttaaatcagaccttttaaaaataagcagaaagtgataccgtggacagcctgccaaatgacgtcacaactaaaaatcgatttcttaaaaaaaaaaacacctgacattttactccacaagagatcatcctgctgcctgatgaatacaagaacacttaacacaacccctttgcttttattataagcggtttgaactggtgttccttctcctaagtcgtaacgcaccagttgtcggggtaatccagacaataatagaagtgaatacaaaacaaacgaacaaaatgacgcttattgccagggaacccgcgcaaaagcgccagacaaaaccctacccagcctctctcgcgagtcccagaagacaaagccgggtcccagcaagcactgcggccaggactacacatcccagcatgcaaggcgcacacttctcccagcgggactacgattcccaacatgctctccggacagctggccggcctccccaccatcccggtgtctggggtctctgcaaaaatggggtctgtctccgcccgcccagcctccctttagcgttaaggatcggacaccacctcccggtagtaaattgtgactattccgagttttggagtcattcttcgttgcctgttttattagggcagcaaaacgcctgtggctagggcgggagtgggcggaggcaggtttgagccaatggcacgggagcggccctgtcgctgccatggcaacggcggccgttctccggggaccggctgcccattgggtagaatctttccagaaggctcgagaagaaggaagcggaagtggcacgtggaggggccggtggaggcgccggtgagtaaatgccgcagattctggaaagttctgatcagtgcgatacataaggctgaggaagtgggacctccccttttgggtcggtagttcagcgccggcgccggtgtgcgagccgcggcagagtgaggcaggcaacccgaggtgcggagcgacctgcggaggctgagccccgctttctcccagggtttcttatcagccagccgccgctgtccccgggggagtaggaggctcctgacaggccgcggctgtctgtgtgtccttctgagtgtcagaggaacggccagaccccgcgggccggagcagaacgcggccagggcagaaagcggcggcaggagaagcaggcagggggccggaggacgcagaccgagacccgaggcggaggcggaccgcgagccggccATGTCGGTGGTACCGGTAGAAAAAATGAGT

/*C. elegans*/ *hsp-70* (C12C8.1)::GFP (2011 bp)

ggatccatttgcttccatcacttttgaacaaattcgatttaatctgtttttagtaatgtatttttgaaactgttacaaatttaattacccatttcctgattccatgaactcatcaaaatccattctttgctggtcttctttaaatttttttgcgatagagcagtagaaactgatagtgacgtcatacactgtattttcgttgaaaaacgttgcagctttctgaaatgctcagaaatttataaagtttggtaaaactgctcactgttgctcctttctctttccaaaatgtatccaaataattgaaatcatccgttttcaaaagctcagttcttccagcaagaggcccaattagcaatccattcgacactacaaccgtttctccaggattaactccaatatccaaaggagataaattaatatttttcatacaaaaactctcaatttttgatagaatcgtcatgtcagagtttggtccaacattttcaaaattgaacgagcatggattgtcgttgctccgagagcttgatggatttgggatcagtgcaatcttattatttttctgagatgcaatagatcgaatagctttggtagcaaataaccgatttgatggattctcaaaatcagcaataatccatgttgtaacatcctaaaaattagttcaatttttaattatcacgttttatcaattttaccgcattcgctaaaactgtatcagattcctccgagaaatagtgcacattctttaaaaattgaatagatcttctgaaaacaaactgttcctaatatatttattttgtattaattctaaaaacctgctgatattcaaaaattccttattgttgaatgccgtagtgatcctgaaatacaattttcaataatttttaaacaaaccagaatttttgtgaacctctgattaaggcgttgaataatatccggatttgtctttttctcgaaccaccaacgatcaatttgtacagaatcttcaattagtccatgaagtaatgcaagttggagtcgtgttgtttgtttttgaatattttgtgtcaacgaagattcaaatctttcaattgatgtctgtaaataatagttattttctttaaattcgaaatgtgtacatagtttcaaatgaaaacttgttatccgtaaaaaatgacttgaaagaacgaactcaccacatctaatggatatccgttgagtagaacaagtggtaattgggttagaccagcttcagccacaaatcgactacctttctggaagaattcaaaaattaaatagttgatttcaatatcaccaattttacaacaacaaaagtggacgcttattacttgaatattactgacagctattagaaaatgttccccaaacgcagatttacttattttgaaacaatttattacctataaatcgcgatatgctcatattgcctcggtgagcaccatgggctcatatgaatcgagcatcgggcacaatgcggtcatcgcgaaaacgaatttgtgagtgtgcacactgcagcgattttcgaggcgctatttgaaaagaaaaaattacagccaaacatttttttaaatattcccttgatactctataatttccagtgattcatattaaaatatacattcttctcttgcaaacgttcgatgagtgtttcttaatccagaaagttcctctatggttcctagcttaaccaatccccactagaaacgtcaacaacaaagacctattctagatatttctttccttttccctggcgtaaacatctgtgatgcactaaccaaaaaacagccacaagaacgttcttgcaaaagagaacagagaaaatagcataggcgacccacagagactcaggcagtggaagaactaaaggatggagcaccccttgcatctagtaaattgtagaaggttctagaagatgccagaggaacgtataaaaactcaaatcttatgcagaattttaaccaaacaagaatttttaacttctgctattcaaacgctgaaattaactgaatATGTCTACATGACCGGTAGAAAAAATGAGT

/*C. briggsae*/ *hsp-70* (CBG22024)::mCherry (2008 bp)

ggatccatcagcgcgattttcagaggaaatttcaacgattttaaccatttggtaatcgataatcgattttttgtattttcaaagtttgcgatgatcgccagtgtgggattttcaggattctctgaagcttctgaatccaattccacaaatctcctctcgttgaacgctttgacgattctgaaaaattcaaatttggcgctgaaaattctcaaattgttcttctattacctcatactaactctggttttcagatccggattcgtctcctctttcagccaaaaatcgtcgattttcacagaatcggtgattagacgttggtagagcgcgagttgaagacgtagcgtttgtttttggatattatcaaagagagcggcgtcgaaggattcagatgaggaggtctggaaacaagagggtcagaggatcagaagaatccttagaatccctagaattcagggatctaggaatctgagaatcctgagaatctgggaatcctgagaatctgggaatccacgaatctaagaatctaagaatcttaagaatccaggaatccaggaatctagaaattcagacatccaggaatctaagaatcctaaaaatccaagaatccaggaatcctaaaatcaaggaaccaaccaaatccagtggatatccattgagaagtgcctgtggtcctttaccaagacccgttttcaacaggaattcgtttccattctgtaaaatttggattttaagttttcaaatttcagttttctgaacaattgaagggttactgtaggagaaattttctttaaaaatgagtgctcccaaaagctgaaaagttgcctgaaaattgcatttttaaagttgaaattcggtctgaaattaagtcaaattcgcaatttttggaaggaaaggattactgtagaagtcgaaaggttactgtaggaacgcaatttcaaaaacagcgaaaattgaaattgctgtgagaacagctaattgtggagtactgtagcaaactaaaactgcaataaagatcaaaaaactagcgaaaaatccaccttcgaaatcatcgaatttcgaattactgtagaaaaccaaaaacttcaaaaaaagctaattgggttactgtacctcttgctcctctcaaaaaaaggggaatctgcgttcggggggaatcgaacccccgtcgaatgcttggaaggcatccatgctgaccattacaccacgaacgcatagaacatcttctagaacattccaaagacaatacgaagctgctatctccgatagattatccgttttccttggatgaacaaggaactagaataaaaagaaaatctactttctggtcagctaaaggaaagggaacatctatcggagataacgtcttcgtattgtttatggaatcttctagaagatgttctatgcgttcgtggtgtaatggtcagcatggatgccttccaagcattcgacgggggttcgattccccccgaacgcagagttccctttttgggagaaggaggtacagtaatccagtagattttttggctttgctacagtaaccaaaataggctcattttcaaggggaaatctcgtcgcttgctgaggagtcctactgtacttttaggactgtttttagcatcacaatcattccaattccttcttctagaaccttctgtgctctcttatcctgcagacttcatagctcatagtccacaaaccaacaaaacaactagaacattctagaattcccccttcttcctcactccctaaaaactgcctcgtaaacaacaatccacccctttctctctcgtctctcttgcactctatttctccccaaaaaaacatggggaacgttctggcaaaacgagatagcatagcggtcaacaggagacgcagacaccgagcacgcctctcaaaatgcatctagaaggttctagaaggcgttgtgatcagggctataaaagctaggaggtcagggaaacggtcagaattcagattaaaatttgctactacaaactacaaaaATGTCTTCATGACCGGTAAAAATGGTG
