# Supplementary figures and images for: Functional Conservation of Cis-Regulatory Elements of Heat-Shock Genes over Long Evolutionary Distances
Source: PLoS One. 2011 Jul 25;6(7):e22677. doi: 10.1371/journal.pone.0022677 (PMC3143172; doi:10.1371/journal.pone.0022677)

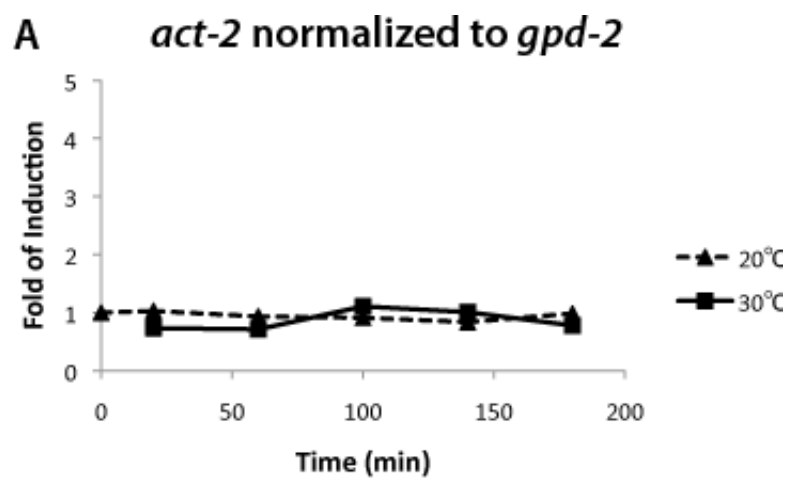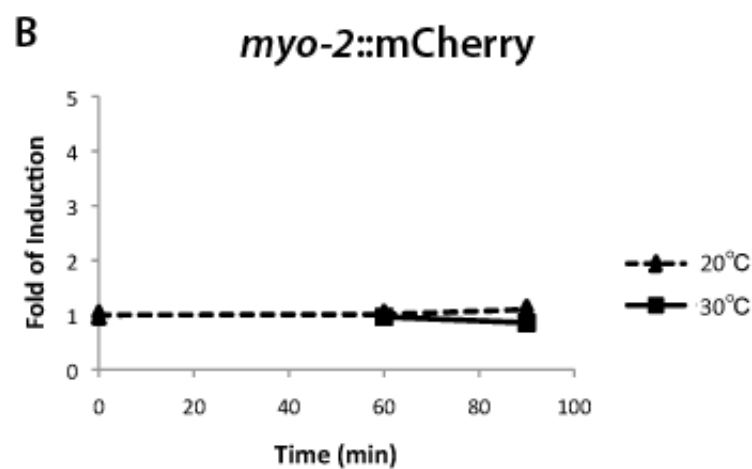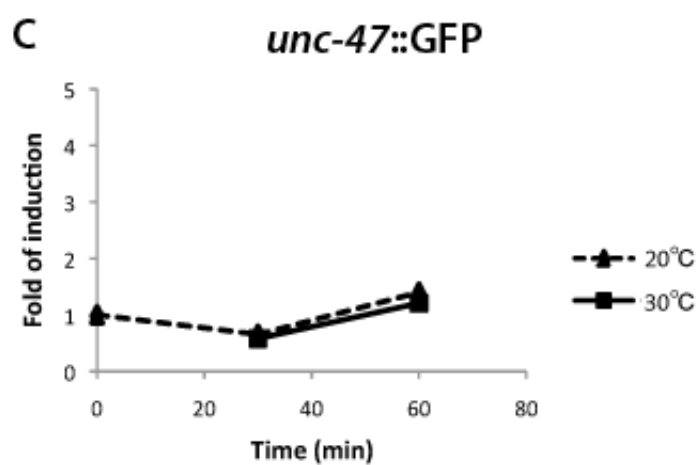

Figure S3

Supplement: Figure S3 — Controls. A) Endogenous expression of C. elegans genes act-2 and gpd-2 is not induced after heat shock. Expression of promoter-reporter gene constructs B) myo-2::mCherry and C) unc-47::GFP is not induced after heat shock. (PDF) [file pone.0022677.s003.pdf]

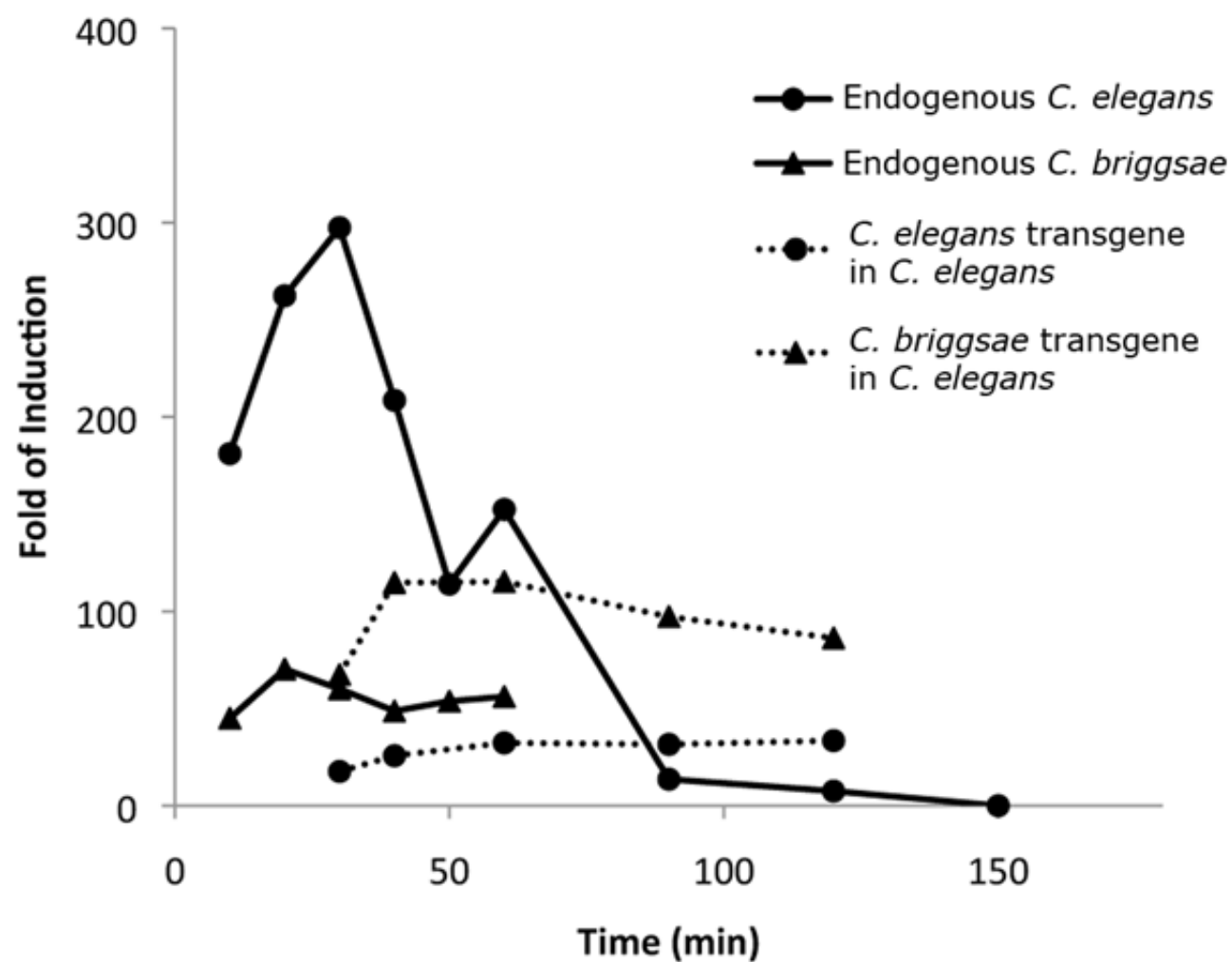

Figure S4

Supplement: Figure S4 — Induction by heat shock of endogenous C. elegans and C. briggsae hsp-70 genes and of transgenic constructs fusing their promoters to GFP. Relative levels of induction were calculated based on the amount of expression just prior to the start of heat-shock treatment. (PDF) [file pone.0022677.s004.pdf]
